# Correction for Inhibition Leads to an Allosteric Co-Agonist Model for Pentobarbital Modulation and Activation of α1β3γ2L GABA_A_ Receptors

**DOI:** 10.1371/journal.pone.0154031

**Published:** 2016-04-25

**Authors:** Alexis M. Ziemba, Stuart A. Forman

**Affiliations:** Department of Anesthesia Critical Care & Pain Medicine, Massachusetts General Hospital, Boston, MA 02114, United States of America; United States Department of Agriculture, Beltsville Agricultural Research Center, UNITED STATES

## Abstract

**Background:**

Pentobarbital, like propofol and etomidate, produces important general anesthetic effects through GABA_A_ receptors. Photolabeling also indicates that pentobarbital binds to some of the same sites where propofol and etomidate act. Quantitative allosteric co-agonist models for propofol and etomidate account for modulatory and agonist effects in GABA_A_ receptors and have proven valuable in establishing drug site characteristics and for functional analysis of mutants. We therefore sought to establish an allosteric co-agonist model for pentobarbital activation and modulation of α1β3γ2L receptors, using a novel approach to first correct pentobarbital activation data for inhibitory effects in the same concentration range.

**Methods:**

Using oocyte-expressed α1β3γ2L GABA_A_ receptors and two-microelectrode voltage-clamp, we quantified modulation of GABA responses by a low pentobarbital concentration and direct effects of high pentobarbital concentrations, the latter displaying mixed agonist and inhibitory effects. We then isolated and quantified pentobarbital inhibition in activated receptors using a novel single-sweep “notch” approach, and used these results to correct steady-state direct activation for inhibition.

**Results:**

Combining results for GABA modulation and corrected direct activation, we estimated receptor open probability and optimized parameters for a Monod-Wyman-Changeux allosteric co-agonist model. Inhibition by pentobarbital was consistent with two sites with IC_50_s near 1 mM, while co-agonist model parameters suggest two allosteric pentobarbital agonist sites characterized by K_PB_ ≈ 5 mM and high efficacy. The results also indicate that pentobarbital may be a more efficacious agonist than GABA.

**Conclusions:**

Our novel approach to quantifying both inhibitory and co-agonist effects of pentobarbital provides a basis for future structure-function analyses of GABA_A_ receptor mutations in putative pentobarbital binding sites.

## Introduction

Pentobarbital (PB) is an intravenous general anesthetic that, like etomidate and propofol, produces its effects in part through γ-aminobutyric acid type A (GABA_A_) receptors [1]. GABA_A_ receptors are pentameric ligand-gated ion channels (pLGICs) that conduct chloride ions when activated, resulting in hyperpolarization and diminished neuronal excitability under typical physiological conditions. Most GABA_A_ receptors in the central nervous system consist of three types of subunits: two α, two β, and a fifth subunit that is usually γ2 in synaptic receptors or δ in extrasynaptic receptors [2]. Each subunit shares a structural motif with the entire superfamily of pLGICs: a large N-terminal extracellular domain (ECD), a transmembrane domain (TMD) consisting of four membrane-spanning α-helices (M1 to M4), and a variable-size intracellular domain between M3 and M4. Typical synaptic GABA_A_ receptors are arranged β-α-β-α-γ counterclockwise when viewed from the extrasynaptic space [3]. This assembly forms four distinct types of subunit interfacial pockets: α^+^–β^–^, α^+^–γ^–^, γ^+^–α^–^, and two β^+^–α^–^, where ‘–‘ corresponds to M1 and ‘+’ corresponds to M3 in the TMD.

R-mTFD-MPAB is a potent barbiturate sedative-hypnotic that photolabels α1β3γ2L GABA_A_ receptors at transmembrane α^+^–β^–^ and γ^+^–β^–^ pockets [4]. Based on sequence alignments, the R-mTFD-MPAB contact residues are homologs of residues photolabeled by etomidate derivatives in the two transmembrane β^+^–α^–^ pockets [5,6]. Both R-mTFD-MPAB and etomidate photolabels are displaced by propofol [4]. PB inhibits photolabeling by both R-mTFD-MPAB and azietomidate, and thus probably modulates and activates GABA_A_ receptors *via* multiple inter-subunit pockets where other intravenous general anesthetics act [4].

Pentobarbital is less potent than etomidate or propofol, but produces similar molecular effects on GABA_A_ receptor activity in voltage-clamp electrophysiology studies. These actions include enhancement of GABA-elicited responses at PB concentrations associated with clinical anesthesia, direct activation (agonism) of GABA_A_ receptors by high PB concentrations, and inhibition by high PB concentrations [7–10]. Quantitative analyses of GABA modulation and direct activation by etomidate and propofol are consistent with formal Monod-Wyman-Changeux allosteric co-agonist models. Indeed, quantitative analyses based on this class of model predicted two equivalent etomidate sites [11] and more than two propofol sites [12] per α1β2γ2L GABA_A_ receptor, consistent with subsequent photolabeling [5,13]. The overall goal of the experiments described here was to generate a quantitative model for pentobarbital co-agonism in α1β3γ2L GABA_A_ receptors. However, in comparison to etomidate or propofol, estimates of PB agonist site stoichiometry, potency, and efficacy are obscured by PB inhibition of GABA_A_ receptors, which occurs at similar concentrations. Here, we describe a novel approach for quantitative deconvolution of PB agonism and antagonism from pseudo-equilibrium voltage-clamp electrophysiological measurements, and model-based analysis of PB co-agonism in α1β3γ2L GABA_A_ receptors.

## Methods

### Animals

Female *Xenopus* frogs were housed and maintained in a veterinarian-supervised facility with temperature regulated at 17 to 19°C, 12 hour light/dark cycles, and fed with frog chow three times per week. Frogs were used as a source of oocytes in strict accordance with the recommendations in the Guide for the Care and Use of Laboratory Animals of the National Institutes of Health. Approval for animal use in this study was obtained from the Massachusetts General Hospital Institutional Animal Care and Use Committee (protocol #2005N000051). Frogs were anesthetized in tricaine prior to *Xenopus* oocytes harvest. All efforts were made to minimize animal suffering.

### Materials

Pentobarbital, salts, and buffers were purchased from Sigma-Aldrich (St. Louis, MO, USA) Pentobarbital was dissolved in ND96 electrophysiology buffer (see below) and pH adjusted to 7.5 on the day of use.

### GABA_A_ Receptor Expression in *Xenopus* Oocytes

Oocytes were prepared as previously described [14]. Complementary DNAs encoding human α1, β3, and γ2L GABA_A_ receptor subunits were subcloned into pCDNA3.1 expression vectors (Thermo Fisher Scientific, Waltham, MA, USA). Messenger RNAs were synthesized on linearized DNA templates using mMessage Machine kits (Ambion Thermo Fisher), purified, mixed in a ratio of 1α:1β:5γ, and diluted in RNAase-free water to 1 ng/nl. Oocytes were injected with 25 ng total RNA mix and incubated in ND96 buffer (see below) supplemented with ciprofloxacin (2 mg/ml) and amikacin (100 μg/ml) at 17°C for 48 to 72 hours before electrophysiological studies were performed.

### Two Electrode Voltage-Clamp Electrophysiology

Experiments were performed at room temperature (21 to 23°C). Oocytes were positioned in a custom-built low volume (30 μl) flow-cell and impaled with two pulled borosilicate glass electrodes filled with 3 M KCl (resistance < 1 MΩ). Electrophysiology buffer was ND96 (in mM: 96 NaCl, 2 KCl, 1 CaCl_2_, 0.8 MgCl_2_, 1 EGTA, 10 HEPES, pH 7.5). Oocytes were voltage-clamped at -50 mV (model OC-725C, Warner Instruments, Hamden CT, USA). Superfusion solutions based on ND96 were selected and delivered from 8 reservoir syringes *via* electrical pinch-clamps (VC-8, Warner Instruments), and a low-volume (< 1 μl) PTFE manifold (MP-8, Warner Instruments) at a flow-rate of 2–3 ml/min. Experiments were coordinated with digitized recording of voltage and current signals *via* a digital input/output interface and software (Digidata 1322 and pClamp 8.0, both from Molecular Devices, Sunnyvale, CA). Currents were filtered at 1 kHz and digitized at 100 Hz, then stored on a computer disk for offline analysis.

For GABA concentration-response studies, voltage-clamped oocytes were exposed to solutions containing GABA (range 0.3 μM to 1 mM) with or without 236 μM PB for 20 s, followed by washout in ND96 for 5 minutes. Normalization sweeps using 1 mM GABA alone were performed every 15 to 20 minutes. Concentration-responses for PB direct activation were performed similarly, using 20 s drug applications and 5 min washouts. Normalization during these experiments was to 1 mM GABA delivered *via* tubing that bypassed the manifold, in order to prevent co-application of PB with GABA.

PB inhibition was studied using a single sweep “notch” experiment where maximal GABA_A_ receptor activation was first achieved by exposing oocytes for 10s to a control solution containing 1 mM GABA plus 100 μM PB (10 s), which maximally enhanced responses relative to GABA alone, with minimal inhibition. This initial activation was followed by 10 s of a test solution containing 1 mM GABA plus high PB (ranging from 300 μM to 3 mM), and another 10 s in the control solution (1 mM GABA + 100 μM PB), and finally ND96 washout for 5 min. PB concentrations for notch experiments were chosen to match those used for direct activation.

### Data Analysis

Digitized data was corrected for baseline leak currents and digitally filtered (10 Hz, Bessel function) using Clampfit 9.0 software (Molecular Devices). Peak currents were normalized to 1 mM GABA controls, and combined normalized GABA concentration-response data (from > 5 oocytes at each concentration) was fitted with a logistic equation using Prism 5.0 (GraphPad Software Inc, La Jolla, CA):

$$I/I_{norm}=F_{max}/(1+10^{\left(LogEC50-Log[GABA]\times nH\right)})$$ (1)

Where F_max_ is the maximal response relative to GABA, EC_50_ is the half-maximal activating GABA concentration, and nH is the Hill slope.

PB direct activation sweeps at 1 mM and higher concentrations displayed early peaks followed by a drop in current amplitude due to PB inhibition, and a “tail” current after discontinuing PB exposure. After baseline correction, we measured both the initial peak amplitude and the maximally inhibited “trough” current just prior to the tail. These were normalized to 1 mM GABA responses in the same oocytes.

Notch inhibition data was analyzed using an approach that corrected for desensitization during the experiment [15]. After baseline correction and filtering, data was imported into Origin 6.1 (OriginLab Corp, Northampton, MA) and plotted. The two control phases of the current sweeps were fitted with either an interpolated straight line or exponential curve, which was overlaid on the sweep. A vertical line was drawn at the point of maximal steady-state inhibition. Both the inhibited current and the interpolated simultaneous “control” current were measured using the data reader tool in Origin, and the normalized “notch” current calculated from the ratio of these measurements. Repeated measurements (n = 6) at each PB concentration were fitted to an inhibitory logistic function similar to Eq 1, constrained to reach 0 at high PB concentrations.

Correction of PB direct activation for inhibition was performed using the steady-state “trough” current activated by PB alone and the PB inhibition assessed with notch inhibition experiments (1 mM GABA plus PB). To calculate the corrected direct activation, we divided the normalized PB “trough” current by the average fractional notch inhibition in the presence of GABA and the same PB concentration. Errors were propagated using standard methods [16]. Corrected PB direct activation results were then fitted with a logistic function (Eq 1). Finally, the fitted PB inhibition logistic function and the fitted PB direct activation logistic function were multiplied together to generate a biphasic concentration-response curve for visual comparison to normalized PB trough current data.

### Monod-Wyman-Changeux Allosteric Co-Agonist Model Fitting

Allosteric co-agonist modeling was performed as previously described [14,17]. Briefly, we combined average normalized peak data from GABA-concentration responses in both the absence and presence of PB with corrected PB direct activation data, and converted these to estimated P_open_ values by renormalizing to the estimated maximal efficacy of GABA (0.83, based on PB enhancement of the maximal GABA response). Estimated P_open_, as a function of [GABA] and [PB] was fitted by non-linear least squares (Origin 6.1) with Eq 2, which describes an MWC co-agonist model with two equivalent GABA sites and a variable number (n) of equivalent PB sites.

$$P_{open}=\frac{1}{1+L_{0}\times {\left(\frac{1+[GABA]/K_{G}^{}}{1+[GABA]/cK_{G}^{}}\right)}^{2}{\left(\frac{1+[PB]/K_{PB}^{}}{1+[PB]/dK_{PB}^{}}\right)}^{n}}$$ (2)

L_0_ in Eq 2 is a dimensionless basal equilibrium gating variable, approximately P_0_^-1^. Initially, L_0_ was set at 25,000, based on previous estimates for α1β2γ2L GABA_A_ receptors [11,18]. However, this resulted in poor fits and we allowed L_0_ to vary, resulting in significantly improved fits based on Chi-squared. K_G_ and K_PB_ are dissociation constants for GABA and PB interactions with closed receptors, and c and d are single site efficacy parameters for GABA and PB, respectively, representing the ratios of dissociation constants in open vs. closed receptors.

### Statistics

Data in text and figures are mean ± sem, unless otherwise identified. Parameters from non-linear least squares fits to Eq 2 are reported as mean ± standard error. Fitted logistic EC_50_s and IC_50_s are reported as mean with 95% confidence intervals. For comparison of peak responses to GABA vs. GABA plus different PB concentrations, we used ANOVA with Dunnett’s multiple comparisons test (Graphpad Prism).

## Results

### Pentobarbital Modulation of GABA-mediated activation

We first studied the modulation of apparent GABA potency by a PB concentration (236 μM) that is equipotent to 3.2 μM etomidate in a standard animal model (2 x EC50 for *Xenopus* tadpole loss-of-righting reflexes). As observed with other general anesthetics, PB dramatically enhanced peak voltage-clamp currents responses to low GABA in oocytes expressing α1β3γ2L GABA_A_ receptors (Fig 1). With maximally activating GABA concentrations, PB increased peak responses by 23 (± 3.0)% and small tail currents were observed after discontinuation of PB plus GABA application. We also observed that 236 μM PB alone activated GABA_A_ receptors, eliciting currents that were 8 ± 3% of maximal GABA. PB at 236 μM co-applied with GABA produced a large leftward shift in GABA concentration-responses (Fig 1, bottom), reducing EC_50_ twenty-fold from 22 μM (95% C.I. = 19 to 27 μM) to 1.1 μM (95% C.I. = 0.8 to 1.4 μM).

**Fig 1:**
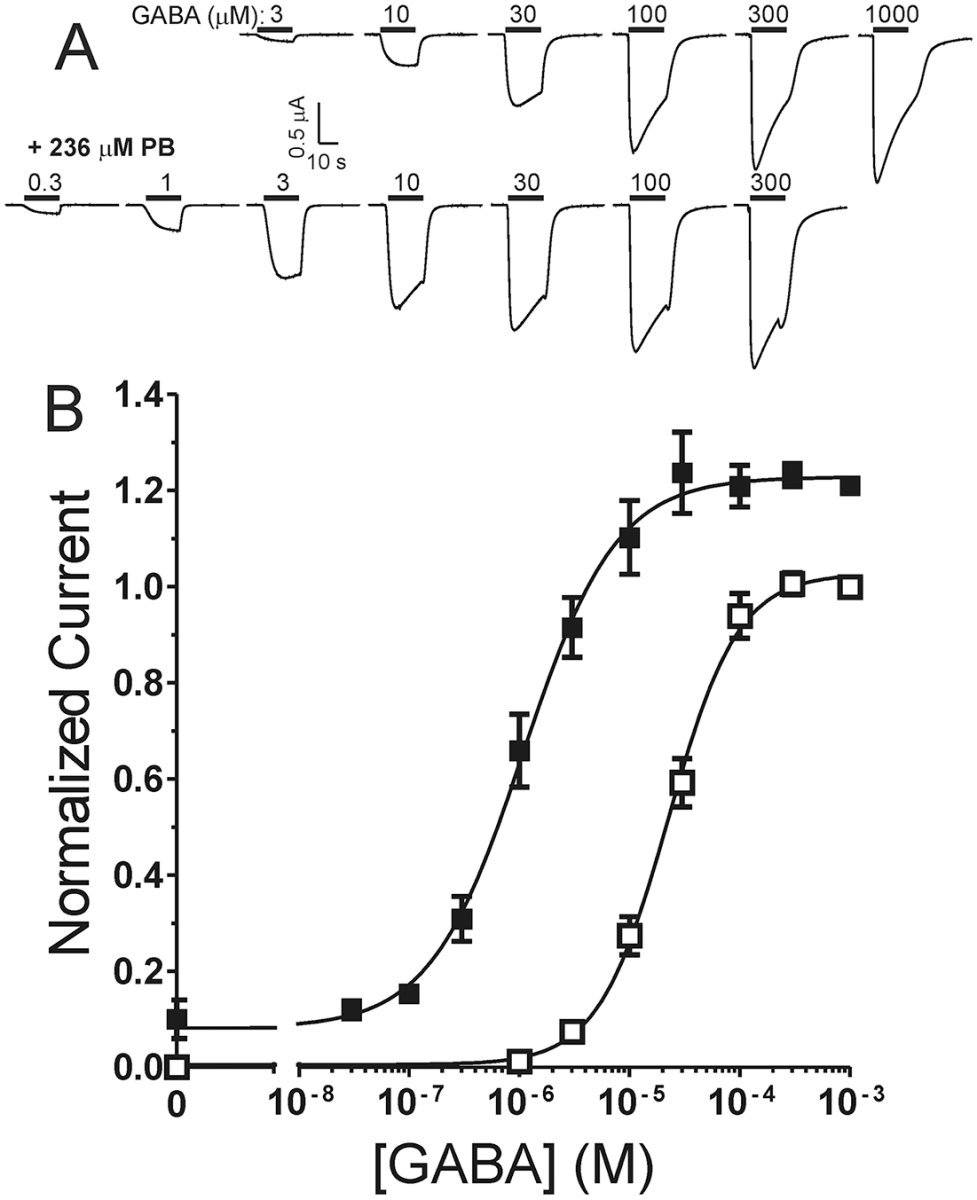
Pentobarbital shifts GABA concentration-responses leftward. ***A)*** Traces are from a single oocyte expressing α1β3γ2L GABA_A_ receptors. Bars over traces represent GABA application with concentration labeled in μM. The lower set of traces were activated by GABA supplemented with 236 μM PB. ***B)*** Combined normalized (to 1 mM GABA response) peak current results from all oocytes (n ≥ 5) is plotted as mean ± sem. Open symbols represent responses to GABA alone and solid symbols represent responses to GABA + PB.

### Pentobarbital Direct Activation

As previously reported, PB directly activated oocyte-expressed GABA_A_ receptors in a concentration-dependent manner. Maximal peak currents elicited with PB at 1 to 2 mM were less than 40% of the maximal GABA response (Fig 2) and no tail currents were observed with under 1 mM PB. PB concentrations of 1 mM and above elicited multiphasic currents with an early peak and a slow inhibitory phase, followed by tail currents after PB application ceased. Early peak currents (Fig 2 bottom, blue squares) showed a biphasic [PB]-dependence with a maximum at 2 mM, while the inhibitory phases grew deeper and tail currents grew more pronounced as PB concentration rose above 1 mM. Thus, PB “trough” currents (measured at the bottom of the inhibitory phase) normalized to maximal GABA peaked at 1.5 mM and steeply dropped at higher concentrations (Fig 2 bottom, black triangles).

**Fig 2:**
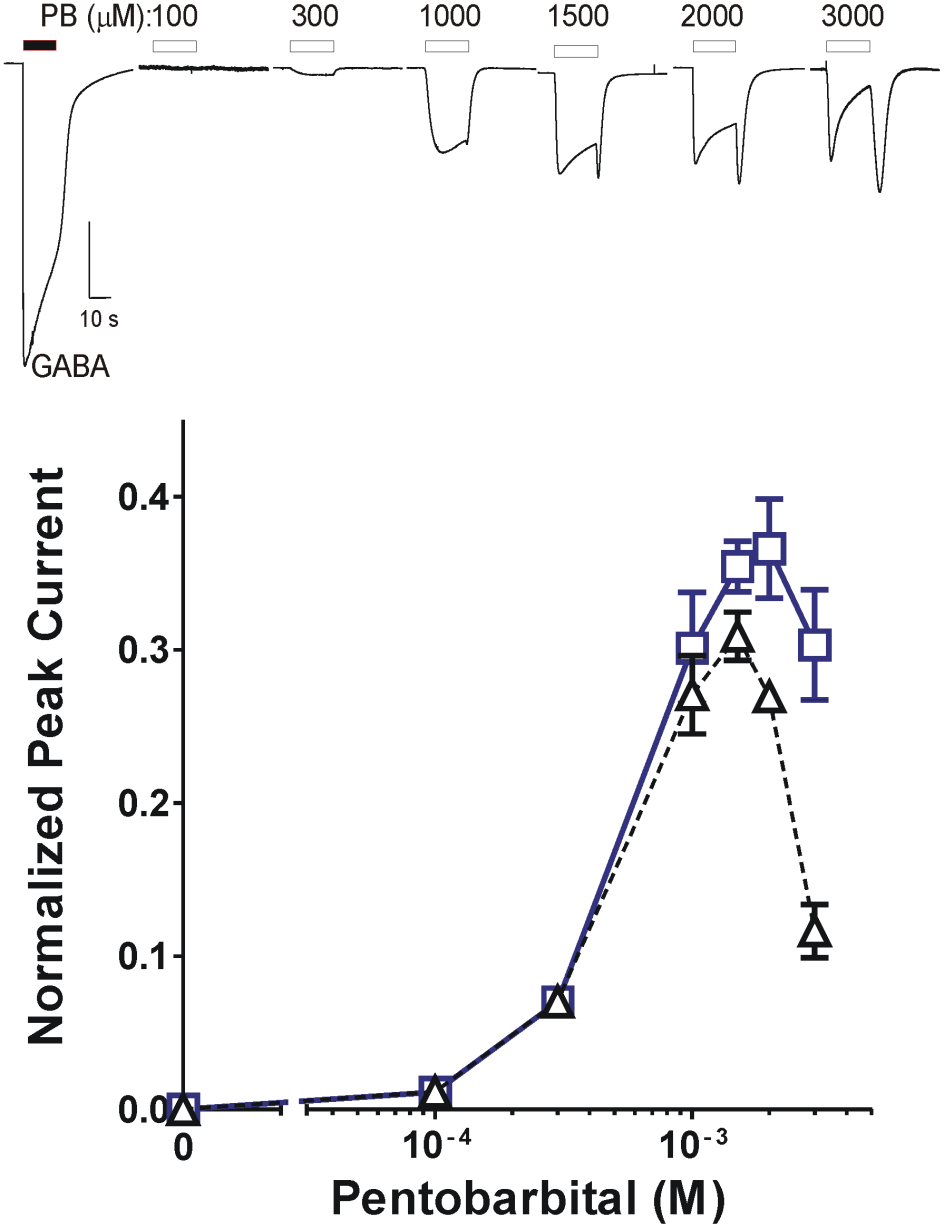
Pentobarbital directly activates and Inhibits α1β3γ2L GABA_A_ receptors. ***Top)*** Traces are from a single oocyte expressing α1β3γ2L GABA_A_ receptors. The first trace is the response to 1 mM GABA (solid bar above trace). Other traces were elicited with PB applications (open bars above traces) at concentrations labeled in μM. At PB concentrations above 1 mM, traces develop “tail” currents immediately after discontinuation of PB exposure. ***Bottom)*** Both early peak PB-elicited currents and the “trough” currents just prior to the “tail” were normalized to 1 mM GABA controls. Mean (± sem, n = 5) results are plotted. Squares represent peak currents and triangles represent trough currents.

### PB-dependent inhibition in GABA-activated receptors

The current responses to PB alone demonstrate a mix of drug-dependent activation and inhibition, which would introduce large errors into analysis of allosteric co-agonist models that account only for activation and positive modulation of GABA-elicited responses [11]. However, the mechanism of PB inhibition is unknown, and no known mutations selectively eliminate this effect. We therefore developed a strategy to quantify PB-dependent steady-state inhibition of GABA_A_ receptor currents independent of gating enhancement, in order to correct mixed activation/inhibition data and reveal the underlying PB-dependent activation. To minimize gating enhancement in our inhibition experiments, we studied activation by maximally activating (1 mM) GABA co-applied with low PB concentrations, seeking a mixture that activated all receptors with minimal inhibition. A combination of 1 mM GABA plus 100 μM PB enhanced peak responses relative to GABA alone by 18 ± 4.4% (p < 0.001 comparison to GABA alone). These currents desensitized slowly and showed minimal tail currents (Fig 3). In comparison, co-applying 1 mM GABA with 500 μM PB produced an early peak comparable to GABA alone followed by a substantial inhibitory phase and large tail current.

**Fig 3:**
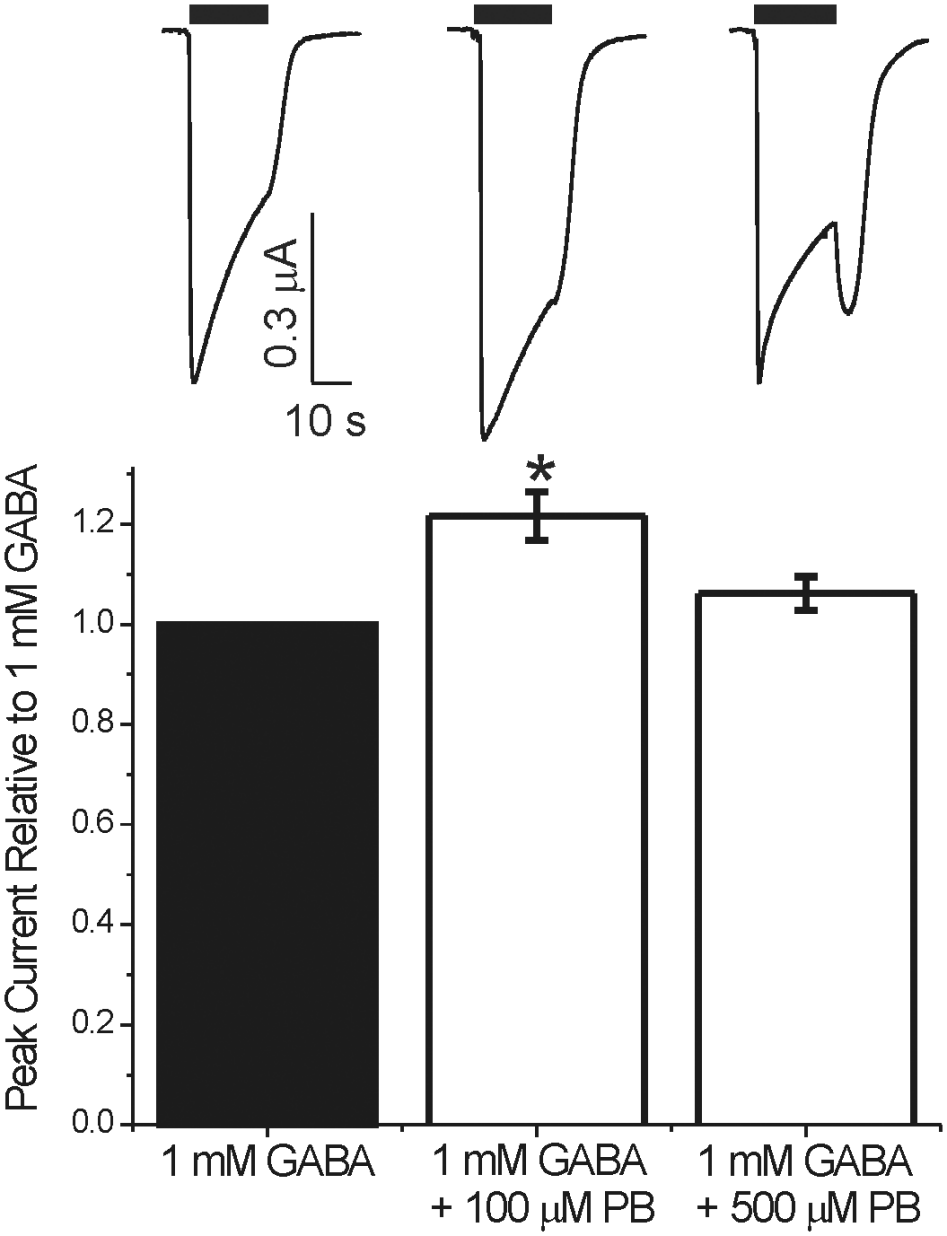
Identifying a PB concentration that maximally enhances without inhibiting currents elicited with 1 mM GABA. ***Top)*** Traces are from a single oocyte expressing α1β3γ2L GABA_A_ receptors. The left trace was elicited with 1 mM GABA, the middle trace with 1 mM GABA + 100 μM PB, and the right trace with 1 mM GABA + 500 μM PB. Note the absence of significant tail current with 100 μM PB and the large tail current with 500 μM PB. ***Bottom)*** Peak currents elicited with 1 mM GABA + 100 μM PB (n = 4) are 18 ± 4.4% (mean ± sd) larger than controls (p < 0.001, one way ANOVA). Peak currents elicited with 1 mM GABA + 500 μM PB are similar to controls. Based on these results, we chose 1 mM GABA + 100 μM PB as the control conditions for notch inhibition studies.

Using 1 mM GABA + 100 μM PB as a maximally activated control condition, we performed single-sweep “notch” experiments where steady-state inhibition by 1 mM GABA combined with high PB could be normalized to an interpolated control current that corrected for desensitization (Fig 4A). PB-dependent steady-state inhibition was well-fit (R^2^ = 0.98) by a logistic function with IC_50_ = 1.13 mM and Hill slope = 1.5 (Fig 4B).

**Fig 4:**
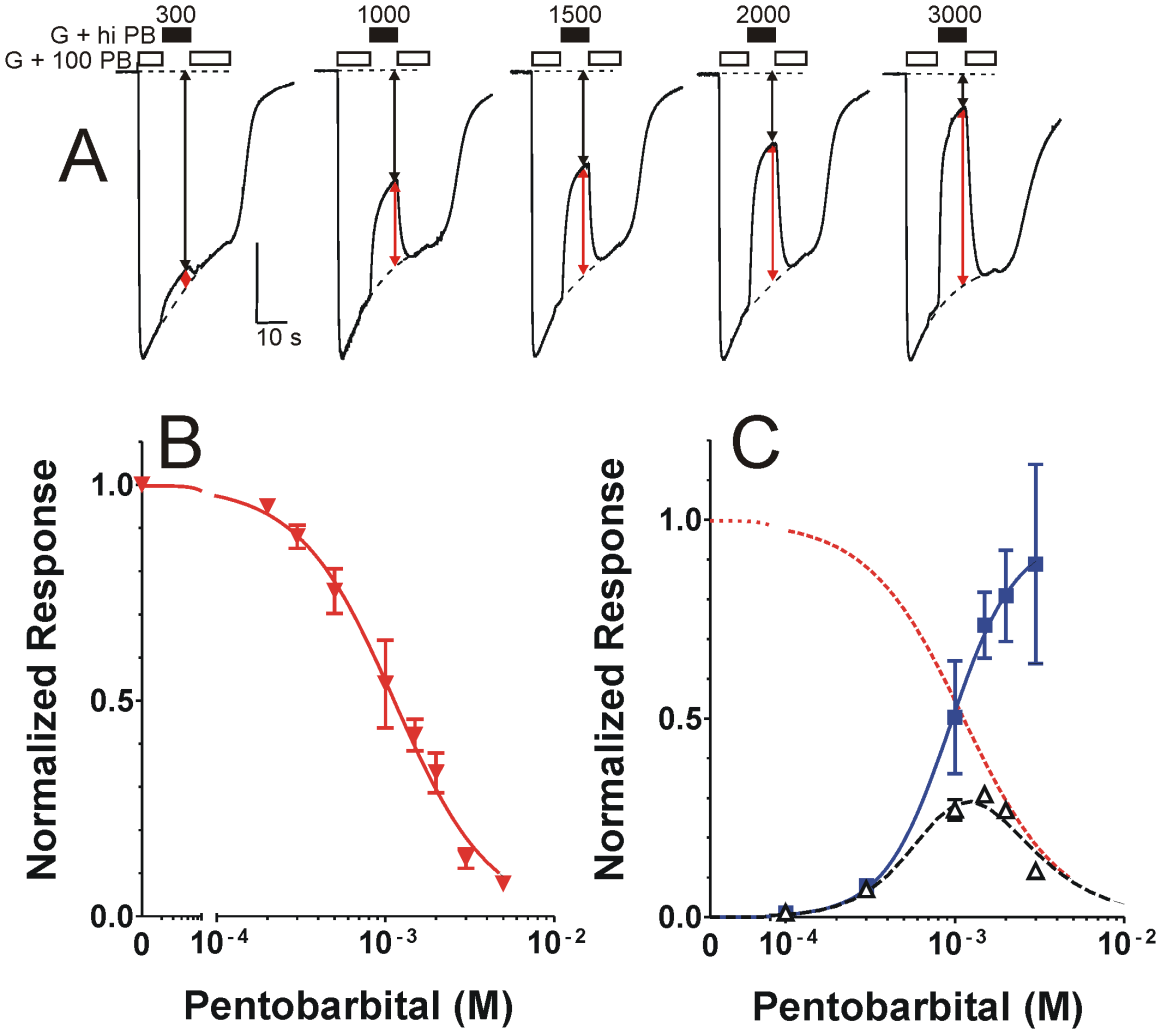
PB “notch” inhibition and correction of PB activation. ***A)*** Traces are from a single oocyte expressing α1β3γ2L GABA_A_ receptors. Bars above the traces indicate exposure to 1 mM GABA + 100 μM PB (open bars) and 1 mM GABA + high PB (solid bars, concentration indicated in μM). Dashed lines indicate both baselines and interpolated curves fitted to the control phases of the traces. Black vertical arrows represent the inhibited current measured at steady-state inhibition, and the combined black + red vertical arrows represent the interpolated maximal activation current used to normalize steady-state inhibition. ***B)*** Combined normalized data from notch experiments (mean ± sd, n ≥ 5 at each concentration) is plotted against [PB]. The line through data represents a logistic fit: IC_50_ = 1.13 mM (95% confidence interval = 1.03 to 1.24); Hill slope = 1.53 ± 0.088. ***C)*** Normalized trough values from Fig 2 (open triangles) were divided by fractional inhibition data from Fig 4B, resulting in corrected PB activation data (solid blue squares). Error bars represent propagated standard deviations. A logistic fit (Eq 1) to the corrected PB activation data (normalized to 1 mM GABA response) is plotted as a solid blue line: Maximum = 0.96 ± 0.092; EC_50_ = 0.94 mM (95% confidence interval = 0.73 to 1.2 mM); Hill slope = 2.2 ± 0.53. Multiplying the PB-dependent activation function x the PB-dependent inhibition function (dashed red line) generates a biphasic dose response (dashed black line) that fits the original steady-state PB activation (trough) data.

### Deconvolution of PB agonism and inhibition

To deconvolve PB agonism from the mixed activation/inhibition in PB direct activation studies, we corrected the normalized PB-induced trough currents (Fig 2) by dividing by the fractional steady-state inhibition observed in maximally activated receptors at the same PB concentration. The corrected results (normalized to maximal GABA response) revealed a monophasic dependence on [PB] (Fig 4C, blue squares). Propagation of errors in these calculations resulted in large uncertainty at high PB concentrations that were associated with > 50% inhibition. A logistic fit (Eq 1) to the corrected data (Fig 4C, solid blue line) resulted in agonist EC_50_ = 0.94 mM, Hill slope = 2.2, and maximum = 0.96. To check for internal consistency of the deconvolved PB actions, we multiplied the fitted logistic functions for PB-dependent agonism (Fig 4C, blue line) and PB-dependent inhibition (Fig 4C, red dotted line), resulting in a biphasic PB response curve (Fig 4C, black dashed line) that closely matched the normalized steady-state trough data.

### MWC Model Fitting

A MWC co-agonist model with 6 free parameters was fitted using non-linear least squares to an estimated P_open_ data set containing corrected PB direct activation and GABA concentration-responses in the absence vs. presence of 236 μM PB (Fig 5; P_open_ was calculated by renormalizing to maximal GABA efficacy = 83%). The model fitted data closely (R^2^ = 0.99) with parameters reported in the legend to Fig 5. The fitted L_0_ is lower than we have previously estimated for α1β2γ2 receptors [11,18], and consequently, the efficacy of GABA (c) also differs from prior estimates. The fitted value of n suggests approximately 2 equivalent PB sites. However, fitted parameters for both PB affinity for resting receptors (K_PB_ = 5.3) and efficacy (d = 0.0026) have errors that are larger than their values.

**Fig 5:**
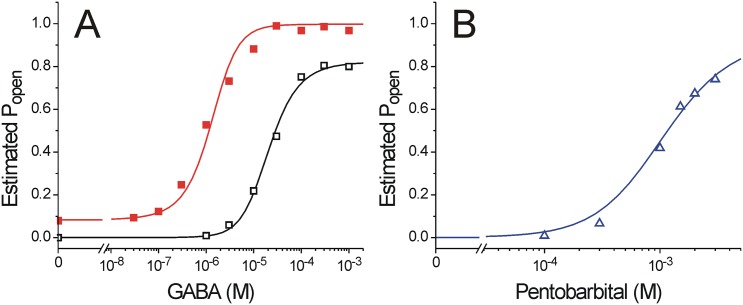
A Monod-Wyman-Changeux co-agonist model for PB activation and GABA modulation in α1β3γ2L GABA_A_ receptors. Estimated P_open_ values were generated from average data in Figs 1B and 4C and fitted with a function describing MWC co-agonism with two equivalent GABA sites and n equivalent PB sites (Eq 2 in Methods). Fitted values were: L_0_ = 1100 ± 460; K_G_ = 33.6 ± 6.2 μM; c = 0.014 ± 0.0035; K_PB_ = 5.3 ± 8.6 mM; d = 0.0026 ± 0.0061; n = 1.7 ± 0.45. ***A)*** Estimated P_open_ values derived from Fig 1B are shown (open symbols are responses to GABA alone and solid symbols are GABA supplemented with 236 μM PB). Lines through data represent the fitted MWC model. ***B)*** Estimated P_open_ values derived from Fig 4C (corrected PB activation responses) are plotted as open triangles. The line through data points represents the fitted MWC model.

## Discussion & Conclusions

The main goal of our current experiments was to develop an equilibrium allosteric co-agonist model for PB activation and modulation of typical synaptic α1β3γ2L GABA_A_ receptors. Similar models for both etomidate and propofol have provided insights into the numbers of co-agonist sites and a robust interpretive framework for structure-function studies [18,19]. An additional challenge presented by PB is that it produces significant inhibition of GABA_A_ receptor currents in the same concentration range as direct activation [7–9]. Therefore, we developed a novel approach to quantify PB-dependent steady-state inhibition and “correct” PB-elicited steady-state currents (Fig 4), which we then used to establish an allosteric co-agonist model for PB (Fig 5). Our results are consistent with the presence of more than one PB inhibitory site characterized by IC_50_ ≈ 1 mM. The fitted parameters for our PB allosteric co-agonist model are most consistent with two co-agonist sites that bind PB with about 5 mM dissociation constants in resting receptors and about 400-fold greater affinity (14 μM dissociation constant) in activated receptors. Our model also indicates that PB agonist efficacy (≈ 0.94) is comparable to and perhaps greater than that of GABA (≈ 0.83).

### Mixed agonist-antagonist effects of PB in GABA_A_ receptors

The mixed agonist-antagonist effects of PB at high micromolar and millimolar concentrations have been described previously, and earlier estimates of the relative affinities of PB for its agonist and antagonist sites have varied, depending on receptor subunit composition, cellular expression system, electrophysiological methods, and analytical strategy. Thompson et al [10] studied GABAA receptors with various human subunit compositions in Xenopus oocytes, and reported PB agonism in α1β3γ3L receptors characterized by EC50 ≈ 190 μM, efficacy ≈ 75% of GABA, and Hill slope ≈ 1.7. A logistic fit (not shown) to our direct activation peak data (Fig 2, open circles) results in EC50 ≈ 590 μM, efficacy ≈ 40% of GABA, and Hill slope ≈ 2.2. The differences in these PB potencies are partially due to different buffer pH (7.0 vs. 7.5) affecting the fraction of protonated neutral drug (pKa = 8.1) that can penetrate membranes to reach the anesthetic co-agonist sites on GABAA receptors (see below).

Many studies of PB actions have used GABA_A_ receptors expressed in HEK293 cells and studied with patch-clamp electrophysiology. Akk & Steinbach [7] used single channel analysis of α1β2γ2L currents elicited with PB. Our equilibrium modeling agrees well with their kinetic analysis, which indicated two PB agonist sites with dissociation constants (K_d_) near 2 mM, and PB agonist efficacy ≈ 0.83. Akk & Steinbach also concluded that there are multiple PB inhibitory sites with IC_50_ (K_block_) between 0.5 and 1 mM. Serafini et al [20] studied α1β3 receptors and developed a kinetic model with two PB agonist sites (K_d_ ≈ 8 and 20 mM, efficacy ≈ 0.5) and two inhibitory PB sites (K_block_ ≈ 0.7 mM). Krampfl et al [8] analyzed data from α1β2γ2L receptor whole-cell currents using a kinetic scheme with one agonist site (K_d_ ≈ 100 mM; efficacy ≈ 0.99) and one open-channel block site (K_block_ ≈ 1 mM). Gingrich et al [9] studied α1β2γ2 receptors, and noted that PB-induced tail current kinetics and amplitudes relative to the early PB-activation peaks differed in whole cells and excised patches. A kinetic scheme that reconciled these observations included two PB agonist sites (K_d_ ≈ 3.4 mM, efficacy ≈ 0.84), 3 PB blocked states (K_block_ ≈ 1.4 mM) and a lipophilic compartment, presumably the cell membrane and cytoplasm, that slows PB exchange rates in whole-cells.

Interestingly, other than one study based on single-channel analysis [7] and an oocyte study that did not quantify inhibition [10], analyses of steady-state PB agonism in GABA_A_ receptors have been based on the amplitudes of tail currents [8,9,21]. This approach is unsuitable for steady-state model analysis, because peak tail currents emerge while PB concentration is rapidly falling, and certainly not at steady-state. Our results show that tail currents recorded in oocytes may be higher than peak currents induced during PB application (Fig 2), but tail currents significantly underestimate PB agonism after correction for inhibition (Fig 4). Indeed, our conclusion that PB is a more efficacious agonist than GABA is consistent with some previous studies [22]. Our novel approach, which applies a simple steady-state inhibition correction to pseudo-equilibrium PB activation results, leads to PB binding and efficacy estimates that agree best with the sophisticated single-channel kinetic analysis of Akk & Steinbach [7] and the rapidly-perfused patch recordings and kinetic analysis of Gingrich et al [9].

### Mechanisms and sites of PB agonism

Co-agonist model analysis indicates that the mechanism underlying PB modulation and direct activation in GABA_A_ receptors is similar to those for propofol and etomidate. Earlier co-agonist model analysis of etomidate suggested two equivalent co-agonist sites [11], and that for propofol indicated 2.6 equivalent co-agonist sites [12]. Photolabeling with analogs of etomidate identified residues located in β-M3 and α-M1 transmembrane helices, adjacent to the two β^+^–α^–^ inter-subunit interfaces [5,6]. There is divergent data on whether these two etomidate sites are equivalent, based on single-site mutations in concatenated subunit assemblies. A mutation in α-M1 produces symmetrical effects in β2-α1 and β2-α1-γ2L constructs [14], while a β-M2 mutation produces asymmetrical effects in γ2-β2 and α1-β2-α1 constructs [23]. The potent barbiturate photolabel R-mTFD-MPAB [24] incorporates in β-M1, α-M3 and γ-M3 helices, indicating binding within both α^+^–β^–^ and γ^+^–β^–^ inter-subunit interfaces [4]. Azi-etomidate incorporates at homologous loci in α-M1 and β-M3 helices abutting the β^+^–α^–^ interfaces. Propofol displaces both R-mTFD-MPAB and azi-etomidate, suggesting it may act *via* four of the five transmembrane inter-subunit pockets [4]. A β-M2 mutation eliminates most of propofol’s modulation [23]. This suggests that the two β^+^–α^–^ sites are high efficacy propofol co-agonist sites. PB displaces R-mTFD-MPAB eight-fold more potently than azi-etomidate, suggesting that PB binds selectively α^+^–β^–^ and γ^+^–β^–^ sites [4]. This is consistent with our current quantitative model analysis, suggesting approximately two equivalent PB co-agonist sites. However, homologous mutations at the 15´ M2 helix positions of α1, β2, or γ2 all affect PB modulation [23], so there may be more than two PB sites with different efficacies in αβγ receptors.

### Mechanisms and sites of PB inhibition

In electrophysiological experiments of GABA_A_ receptors in HEK293 cells and patches as well as *Xenopus* oocytes (Fig 2), PB activation precedes inhibition. High concentrations of PB produce interruptions in single-channel openings as well as a shortening of open-channel burst duration, suggesting that inhibition may involve both channel block and desensitization [7]. However, macro-currents indicate that PB slows desensitization of α1β3γ2L [21]. Most of the kinetic models developed to account for PB agonism and antagonism in GABA_A_ receptors [7–9] are based on inhibition or block of open-channels, and adequately account for both kinetic and equilibrium effects in macrocurrents. These models are also consistent with observations that PB inhibition is greater when GABA_A_ receptors are highly activated, as evident in electrophysiological recordings stimulated with low PB alone versus GABA plus low PB (e.g. PB inhibition in Fig 2 vs. Fig 4). Recently, a convulsant barbiturate photolabel, S-mTFD-MPPB, was reported to photolabel residues in the γ^+^–β^–^ transmembrane interface of α1β3γ2 receptors [25]. Moreover, S-mTFD-MPPB inhibits α1β3γ2, but not α1β3 GABA_A_ receptors, indicating that the γ^+^–β^–^ site is crucial for inhibition [26]. These data suggest that occlusion of the open chloride channel may not underlie inhibition by PB, and suggest an alternative mechanism whereby inhibitory barbiturates bind at an allosteric site, possibly overlapping with the γ^+^–β^–^ allosteric agonist site. Rightward shifts in GABA-response curves also suggest that inhibitory barbiturates act as inverse agonists [26]. It is notable that photolabeling identifies only one inhibitory barbiturate site, while our data and that of most other studies of PB are consistent with multiple inhibitory sites, while occupation of one site inhibits channel conductance.

### Limitations of this study

A significant limitation of this study results from the mathematical correction of deep inhibition, magnifying both steady-state activation as well as its uncertainty. The resulting variances in corrected PB direct activation parameters could limit interpretation of structure-function effects. Furthermore, the large uncertainties in co-agonist model parameters for PB affinity and efficacy emerged from a lack of data at [PB] > 3 mM, the range where maximal agonism, but also maximal inhibition, is observed. The high efficacy of PB agonism evident in the corrected data also increases uncertainty in co-agonist model parameters for PB binding and efficacy, because model sensitivity drops as the fraction of activated receptors approaches 1.0. The mathematical approach we took to correcting PB-dependent activation data for PB inhibition presumes that agonist and antagonist mechanisms are independent. Our approach also presumes that PB inhibition is independent of receptor open probability. This is a feature that was also included in the optimal model identified by Serafini et al [20], but not models that presume that PB inhibition is open-channel dependent [7–9]. Another limitation of this study is that we used a racemic mixture of R-PB and S-PB. While barbiturate inhibition appears to be non-stereoselective [26], there is evidence that purified S(-)-PB is about twice as potent as R(+)-PB in ablating righting reflexes in animals [27] and in enhancing GABA_A_ receptor activation [28]. Independent modeling of the effects of individual enantiomers would likely result in modestly different results.

## Conclusions

We developed a novel experimental approach to electrophysiologically isolate and quantify PB inhibition of GABA_A_ receptors expressed in *Xenopus* oocytes, and used these results to correct steady-state activation data elicited with high PB concentrations. Corrected results for PB activation combined with PB modulation of GABA responses enabled fitting with an allosteric co-agonist model. Our approach provides independent characterization of the stoichiometries, affinities, and efficacies of PB sites mediating both positive modulation and inhibition. These results agree with estimates from some earlier kinetic studies based on patch-clamp techniques. Our new experimental-analytical approach is expected to be useful in studies of the co-agonist vs. inhibitory effects of PB in receptors with mutations in putative PB binding sites.
